# Will a High Preliminary Educational Requirement Remove the Existing Evil?

**Published:** 1902-03-15

**Authors:** G. S. Junkerman


### DENTAL REGISTER.

Vol. LVI. March 15, 1902. No. 3.

### WILL A HIGH PRELIMINARY EDUCATIONAL REQUIREMENT REMOVE THE EXISTING EVIL?

BY G. S. JUNKERMAN, M.D., D.D.S.

Read at the Ohio State Dental Society, December 5, 1901.

The above title presumes the existence of a double hypothesis; that an evil exists, and that a high preliminary education is being used as a germicide to destroy the microbe. We believe the diagnosis to be correct, but the result of the action of the therapeutical agent suggests a change of medicine, or a consultation of physicians. Our patient grows worse from day to day. He is becoming despondent even in the midst of a cheerful and hopeful bearing of his doctors. He seriously considers if it were not best to prepare himself for another world.

Colleges of medicine and dentistry, particularly, have become exceedingly strenuous on the subject of a high preliminary educational requirement for entrance into their course of study. Colleges have not assumed this attitude entirely of their own choice. The pressure has come from the profession at large, aroused by the fear that its family was getting too large and that there would not be sufficient food in its larder for the support of so many offspring. There are two sides to the question, and there has been a considerable spirit of business displayed on both sides: the colleges on the one side fearing the decrease of the number of students to the depreciation of their revenues, and the profession, represented by the examining boards, fearing that its livlihood would be curtailed by an overcrowding of the ranks. There is no doubt that the profession at large is the adjudicator; its moral persuasive power is immense for good or evil. It should be realized that great power carries with it great responsibilities. The question of which was first, the hen or the egg, has never been satisfactorily settled, but that we had dentists before we had dental colleges is a matter of history. The college is the creation of the dentist, and he should have the right to control his own offspring, even to the extent of using the rod, if necessary, in cases of incorrigibility. The rod has been used in the form of a higher and higher preliminary educational requirement. The high-spirited animal will revolt under the whip and may be spoiled by its too frequent or improper use.

If colleges are necessary to the profession (and we presume that they are, or they never would have been created), then they have the right to exist. They can not exist without students, for the profession is not one of sufficient wealth to guarantee endowments and bequests for the support of dental colleges. There are now about fifty reputable dental colleges in this country, and every catalogue of these schools contains but one single requirement for admission, which is some degree of educational attainment and incidentally one hundred dollars. If it should be made incidentally five hundred dollars and no culture, you would all raise your hands and voices in holy horror. Some would cry "class legislation." Others would have visions of the approach of a horde of uncultured Cossacks sweeping down upon them to snatch away the crust of bread so necessary to their existence. We believe that both of the above phases are strictly and purely class legislation, and of the two the high financial preliminary requirement would more nearly correct the evil than the other. In making this statement we fully realize the advantage of education in any calling, and that even the hod-carrier would pack bricks and mortar better if he knew something of algebra and geometry. Of what avail, however, would be his knowledge of higher mathematics if he had rickety bones or curvature of the spine. If education of all kinds is a benefit in any calling, will not three or four years in the dental college benefit a man? Should you have the right to prevent study or learning of any kind? You may be right when you prevent a man from practicing dentistry, but can never be right when you say he shall not study it. Throw open the wide gates—let everybody in. Give them a chance, no matter how desperate their case may be. There will be a large number to pick from. If they can not stand the rigors of the campaign they will be no worse, but better, for the trial. Students under these circumstances know, at least, that they are on trial and success rests on their own endeavors. As the matter stands now, their grading at school is the passport that opens the gate. They may be lame and halt morally and physically, but they enter with a self-consciousness of having fulfilled every requisite. Others have the door slammed in their faces to the detriment, many times, of the profession. The entrance gate wide open, the exit gate narrow and in single file, inspect each soldier. You have plenty to select from, and, if any have a weak spot, turn him down. Colleges would have more students then, without apprehension from the profession. The profession would have less dentists, but they would be better, and it would have more to offer for high quality men, and, with high quality, prestige would be secured.

If a man could afford to pay five hundred dollars yearly tuition, he certainly could not afford to graduate from a dental college and go out and prostitute his profession by operating a cut-rate dental office. Would not this help to remedy that evil? A professional calling obtains prestige only from the quality of its exponents. Have we to-day a sufficient prestige to attract many men who can honor the profession? Eight years in the primaries, four years in the high school, four years in college—we must have a strong magnet and some strong inducements that do not exist in reality (and personality either) in the profession to-day to attract a man who has campaigned through such a long course of schooling, to enter the study of dentistry. All the other professions attract men of such caliber. Does any evil exist?

The experience of the United States Army dental surgeons should be a lesson to the profession. Are the teeth of a soldier of more importance than the teeth of a private citizen? The United States Army Board of Dental Examiners has said that 75 percent, at least, of tbe dentists of our country are not fitted to fill the teeth of a soldier. Will the college men acknowledge or allow that this 75 percent have broken into the dental colleges of our country without having the passport of preliminary educational requirement? This is the only passport demanded.

Preliminary educational requirement will not reduce the number of dentists. It may reduce the number ot dental students. The number to select from will not be so large, and consequently the selection can not be as choice. The rigidity of the army regulation is a travesty upon our admission to practice and a study picture to those who are always crying prestige, dignity and elevated standing for dentistry as a learned profession. We have not taken that position except in the self-conceit of a few who, like the ostrich, bury their heads in the sand and will not see those things that concern the salvation of the profession. We have tried a fairly high preliminary educational requirement, but it won't do; the evil remains and grows a bit. As all good things go by contraries, let us apply such a principle. Open up the entrance gates, but tighten the exits. Let the dental colleges educate. Let an independent commission examine. This will insure honesty on the part of the colleges and perfect protection to the profession.

DISCUSSION.

Dr. L. E. Custer: I would commend Dr. Junkerman for the style of his paper, it is so clear and concise. Papers written for this Society and its consideration are for the good of the profession. We shall not all agree with this paper, perhaps, still it will bring out a good discussion. I believe in elevating the dental profession to a higher standard, and to do this I think the preliminary requirements for the dental student should be raised. I have maintained that from the very first. It has been my practice to say to those who have applied to me to take up work preliminary to their course in college, that I required a degree in letters and five hundred dollars. I have not, consequently, prepared many students for college.

Dr. H. A. Smith: Dr. Junkerman has presented a very concise paper. He advocates a doctrine which was advanced ten or fifteen years ago. Dr. Whittaker, one of the most eminent teachers of medicine, said to me ten years ago, "My opinion is we can not control the entrance of students into a college very well; I believe we should let them all in, and sift them out afterwards." This would have been all right in the hands of Dr. Whittaker, but I question if it would be best to make it the practice of the average school. Going back to an earlier date in the dental education, members of the profession had the preliminary training of students and did what they could to ascertain whether their students were fit subjects for dentists or not. This is not the case to-day, however. The majority of the students come to us without any training in an office and depend entirely for their development upon the college. This makes the colleges to-day responsible for the dentist's election as well as education. The best example of where increased preliminary education is required is in John Hopkins University. Here all the applicants must be a B. of A. They do not say, "or the equal of that," but must have the degree. Medicine is credited as a learned profession, while dentistry is not, although we are getting to be so recognized. In dentistry about 5 or 6 percent of the students have had a liberal education; in medicine, about 8 percent; the legal profession, about 20 percent. The profession of the ministry is composed more nearly of the educated men. Every college professor can point out many men who were very sorry specimens when they first came to the college, but turned out to be very good dentists.

An experienced man in such matters can tell in the conversation or personal examination whether or not the man has it in him to make a good dentist. A man who has a high school education does not always make the best dental student. I do not think we have a right to require an extensively high entrance requirement at present. It is possible to make the standard higher than we have any right to do. The student who comes prepared with the ability to study effectively will experience no trouble.

Dr. H. J. Bosart: I think the colleges would do well to keep a closer tab on their demonstrators, who are usually chosen because they are good in the branch which they teach; but many times their lack of education in other branches is entirely overlooked. I have in mind a college which employed as a demonstrator a man whose partner at that time was an advertising dentist, and they are now both running an advertising office. I know another case where a man was very much below the average of an educated man. If you place a student who may be a graduate from Yale or some other literary school under such a demonstrator, you can imagine his feelings and the influence such an instructor could have with him.

Dr. L. L. Barber: Dr. Smith said he could point to some men who came to the college with a very common education and turned out to be very good dentists. These men would have been very good in any line they followed. The dean of a dental college which has raised its course to four years and increased its entrance requirements to the equivalent of a first-class high school, said to me recently that as a result the class of students they had entered seemed more promising than any previous freshman class in the school's history. This speaks for itself.

Dr. H. A. Smith: I do not think that the class of students that this college has received is due to the raising of the entrance requirements; we are getting a better class of students every year. The four years course will cut off many men who will be unable to attend college on account of expense, and undue length of time is just as much an injustice as anything else. We should educate the student in just as short a time as possible; time is wasted in a four year's course in any dental college. In this course he is required to do a lot of preliminary work which should be done before he reaches college. In reference to demonstrators, some colleges are taking on men who are utterly unfit for their work. To me a position of demonstrator is a very important one. A good demonstrator should receive liberal remuneration; many of them really are earning better pay than the professors.

Dr. Junkerman, in closing the discussion: I regret that this paper came up at such a time as this, when the Association is in a hurry. I believe this Society ought to have spent more time than it has upon the discussion; it is important, it interests us all.

In regard to the army regulation, these 75 percent were estimated on a number which came up for examination before the board, and it must be remembered that these 100 were selected by the highest recommendations that could be gotten. They received letters of invitation to appear before the examination board at the suggestions of some dentists who were high in the profession, and these men selected only those whom they thought capable. So when 100 men appeared before the board they were supposed to be the best qualified men, and out of these 75 percent were turned down, only twenty-five passing. Of course the army regulations require more than the simple mental examination. The applicant has to be perfect in body and of good morals. And by the way, I do not believe any delicate individual has a right to practice dentistry; therefore I do not believe he should be allowed to enter a college. I think all will agree with me in this statement, for every practising dentist realizes that many times our patients are controlled by mere physical contact, as it were, of the dentist. So the army is quite right in taking that into consideration. I think that the university dental schools put more time in their course than is necessary.

I did not advocate by any means a low standard for the dentist; if any one construed my paper so he has misunderstood it. I only claim that it is not always the best literary educated man who will make the best dentist, as I have found in many cases where high preliminary attainments were required they did not make the best dentists. Literary attainments are all right, but they alone are not sufficient. I should like to see as high a preliminary requirement for the dental profession as is exacted by the United States surgeons.

